# Effect of Jackfruit Leaf Extract (*Artocarpus heterophyllus*) on *Sitophilus oryzae* Mortality and Rice Quality

**DOI:** 10.1155/2023/1579432

**Published:** 2023-10-16

**Authors:** Astija Astija, Evi Wardani, Vita Indri Febriani, Fatma Dhafir

**Affiliations:** Biology Education Study Program, Faculty of Teacher Training and Education, Tadulako University, Jl. Soekarno Hatta Km. 9, Palu, Central Sulawesi 94119, Indonesia

## Abstract

*Sitophilus oryzae* is an insect pest known for its destructive impact on rice crops. Chemical pesticides continue to be employed for the prevention of *Sitophilus oryzae*. The aforementioned phenomenon exerts adverse effects, notably in the form of human intoxication. Hence, one alternate approach to address the issue involves utilizing a preparation derived from the leaves of the jackfruit tree. The leaves of the jackfruit tree are known to possess many bioactive compounds such as flavonoids, saponins, and tannins, which have insecticidal properties. Hence, the objectives of this study are to investigate the impact of jackfruit leaf extract on the mortality rate of rice insects and to evaluate the quality of rice. The study was carried out in the Biology Laboratory of the Faculty of Education and Teacher Training at the Tadulako University. The study employed a research strategy known as a completely randomized design (CRD), which included five treatments. Each treatment was repeated in four biological and ten technical replicates. The treatments were as follows: treatment A served as the control and involved the use of *Bestrin forte*, treatment B involved the application of a 10% jackfruit leaf extract, treatment C involved the application of a 20% jackfruit leaf extract, treatment D involved the application of a 30% jackfruit leaf extract, and treatment E involved the application of a 40% jackfruit leaf extract. Every treatment was administered through spraying to all ten insects and that was repeated four times. The data collected were subjected to analysis using analysis of variance (ANOVA) and supported by the SPSS-25 software. The findings of the study indicated that the application of jackfruit leaf extract (*Artocarpus heterophyllus*) at specific time intervals (20th, 40th, and 60th minutes) resulted in a noteworthy impact on the death rate of rice beetles. Furthermore, the extracts successfully preserved the olfactory attributes of the rice, ensuring its quality. Nevertheless, their ability to uphold the standard of the rice in relation to its color and flavor was inadequate. The efficacy of the jackfruit leaf extract in eradicating rice bugs was found to be highest when applied at a concentration of 40%.

## 1. Introduction

Rice is a staple food for Indonesian people. Consumption of rice from-year-to-year has increased along with the increasing population of Indonesia [1–4]. Therefore, the Indonesian government has programmed an increase in rice production to meet the national demand for rice. Lately, that program has been nearly accomplished. However, an important problem faced at this time is regarding postproduction handling, being the problem of handling rice storage.

Stored rice is often damaged by pests. One of them is caused by the presence of rice beetle pests (*Sitophilus oryzae*). Rice beetle is one of the insect pests belonging to the genus *Sitophilus*. Appearance of *Sitophilus oryzae* during rice storage is caused by unfavorable storage conditions such as low temperature and humidity in the warehouse. Under these conditions, the rice beetle can quickly reproduce. Several studies reported that the average daily temperature is in the range of 27–29°C and the average daily humidity is in the range of 75–79% which is a very suitable condition for growth and development *Sitophilus oryzae* [5–7].

Damage to rice results in reduced quality of rice such as rice with small holes so that rice becomes brittle and breaks easily into powder. Efforts to deal with pest attacks from rice beetles that have been carried out are by using chemical insecticides. These insecticides are made from organophosphate, carbamate, or pyrethroid compounds [8–10]. These compounds can be used to suppress damage to rice during storage by killing rice beetles. The use of synthetic insecticides from the organophosphate group in the management of insect pests is highly relied upon by the community. However, they did not realize that the use of these synthetic insecticides had a negative impact on rice such as it can poison humans, causing symptoms of dizziness, nausea, and weakness [11–14]. At least 20,000 people per year die from pesticide poisoning [15, 16].

The search for efforts to control beetle pests that are safe for the environment and human health is still ongoing. One alternative that might be used to deal with this problem is to use natural insecticides. Natural insecticides are easily decomposed in nature so they do not have a negative effect on the environment and do not poison humans. The advantages of using natural insecticides are that they are more environmentally friendly, cheap, and easy to obtain, compatible when combined with the control of other materials, and free from pesticide residues. However, this natural pesticide has several weaknesses, including relatively slow working power, does not kill target pests directly, is not resistant to sunlight, is less practical, does not last long in storage, and sometimes has to be used repeatedly [17–20].

Based on the advantages and disadvantages of using plant materials for insecticides, it is very necessary to find suitable plant materials so that they have more advantages but few weaknesses. Considering that natural ingredients are easy and widely available and have not been utilized optimally, one of them is the use of natural insecticides using jackfruit leaves. The selection and utilization of jackfruit leaves is an alternative solution for handling rice weevil pests and may be able to maintain the quality of rice based on the results of identification of chemical compound groups carried out that jackfruit leaf extract contains metabolites such as flavonoids, saponins, and tannins, that are used for antibacterial, antifungal, larvicide, pesticide, and antioxidants [21–24]. In addition, preliminary tests on jackfruit leaves regarding the potential of jackfruit leaf extract (*Artocarpus heterophyllus*) as a mosquito bio-larvicidal *Culex sp*. that jackfruit leaves contain tannins of 3.08%, flavonoids of 0.92%, and saponins of 1.36% [23]. Saponin substances have a way of working as stomach poisons and inhibit the work of the cholinesterase enzyme in mosquito larvae and flavonoids act as respiratory poisons, causing death in mosquito larvae [25, 26]. Meanwhile, tannins can reduce the activity of digestive enzymes (proteases and amylase) and disrupt the activity of intestinal proteins so that they will experience nutritional disorders [27]. With regard to the reports of the results of these studies, it is possible to apply secondary metabolites from jackfruit leaves to eradicate rice beetles.

Jackfruit leaves as described above can be selected as an alternative natural insecticide. This is because besides jackfruit leaves containing the substances such as saponins, flavonoids, and tannins; so far, no studies have been conducted on insecticides using jackfruit leaves on the mortality of rice beetles. In addition, what is the effect of using jackfruit leaf extract on the quality of the rice? Therefore, the research that was carried out to address the problem regarding damage to rice by rice beetles is how the effect of jackfruit leaf extract (*Artocarpus heterophyllus*) as a natural insecticide against rice beetle mortality (*Sitophilus oryzae*) and rice quality. Thus, this study was conducted to examine the effect of jackfruit leaf extract on the mortality of rice beetles and to examine rice quality. From the results of this study, it is hoped that it can contribute to the handling of rice storage so that the quality of rice remains guaranteed without causing health problems for humans.

## 2. Methods

The study was carried out in the Biology Laboratory, Faculty of Teacher Training and Education, Tadulako University, spanning from December 2021 to March 2022. The research employed a fully randomized design (CRD) comprising of five treatments and four replications. The treatment regimen comprises the following groups: A, which serves as the control and is administered *Bestrin forte*; B, which receives a 10% concentration of jackfruit leaf extract; C, which is given a 20% concentration of jackfruit leaf extract; D, which is administered a 30% concentration of jackfruit leaf extract; and E, which is provided with a 40% concentration of jackfruit leaf extract.

The beetles were acquired from the Animal Husbandry Service of the Regional Government of Palu City. The process of supplying beetles involves the controlled reproduction of both female and male beetles within a confined environment, such as a jar containing rice. There exist discernible differences between female and male beetles, mostly in terms of their physical characteristics. Female beetles may be identified by their thinner and smoother snouts, as well as their comparatively greater body size. Conversely, male beetles possess a broader and rougher snout, along with a lower body size. Rice beetles deposit their eggs into crevices found in seeds or particulate matter. The female rice beetle has a daily oviposition rate of around four eggs. The life cycle of the subject under consideration is around five months, during which it has the capacity to lay a range of 250–400 eggs. The eggs undergo hatching after a period of three days. The larvae use rice as their primary source of sustenance and have a lifespan of 18 days. Subsequently, it undergoes a six-day period of pupation. The rice beetle utilized in this investigation was an adult specimen aged 25 days, as this particular age corresponds to the stage of fertilization.

The simplicia jackfruit leaves were prepared using a total of one kilogram of freshly harvested leaves obtained from Palu City, situated in the Central Sulawesi Province of Indonesia. The leaves are subjected to a comprehensive washing procedure utilizing a continuous flow of water to effectively remove any contaminants that may be present on the samples. The samples underwent a drying process using blotting with a piece of tissue, followed by further cutting into uniform dimensions. Subsequently, the specimens underwent a desiccation procedure within a controlled environment, utilizing an oven maintained at a constant temperature of 60°C for a period of 72 hours. After the completion of the drying process, a dry sorting method was employed to separate the damaged pieces. Following that, the separated pieces were finely ground using a blender, leading to the generation of 463 grams of jackfruit leaf powder, often referred to as simplicia.

The preparation of jackfruit leaf extract involves the maceration procedure with several adaptations [28]. The process of preparing jackfruit leaf extract involves several sequential steps. Initially, the jackfruit leaves are thoroughly washed using clean water. Subsequently, the leaves are air-dried to remove any moisture content. Following this, the dried leaves are finely chopped to reduce the size. A total of 463 g of jackfruit leaves are then placed into an Erlenmeyer flask. To facilitate extraction, 1200 ml of 96% ethanol is added to the flask. The flask is securely closed, and the contents are agitated using a shaker for 3 × 24 hours. After agitation, the mixture is filtered using a filter paper to separate the liquid extract from any solid particles. The resulting filtrate is then subjected to further processing using a rotavapor apparatus. This apparatus aids in the separation of the solvent from the extract by evaporating it in a water bath. As a result, a concentrated extract is obtained. Subsequently, the extract was diluted using distilled water in accordance with the desired concentration for each treatment, namely, at concentrations of 10%, 20%, 30%, and 40%. These concentrations were applied successively as treatments B, C, D, and E. Treatment A acted as a control using *Bestrin forte.*

An experiment was conducted to assess the efficacy of jackfruit leaf extract (*Artocarpus heterophyllus*) in controlling the population of rice beetles (*Sitophilus oryzae*). The experimental procedure involved the application of a 9 ml solution of jackfruit leaf extract by spraying. To start the administration of jackfruit leaf extract, the rice beetle specimens were partitioned into five containers with a jar-like form (refer to five treatments). Every individual container is filled with 200 grams of rice and a population of 10 rice beetles. Subsequently, jackfruit leaf extract was administered in five treatment groups, with a dosage of 3 ml each jar for a single application at specific time intervals of 20, 40, or 60 minutes. The treatments of the experiments were repeated 4 times. The mortality of rice beetles was assessed using the quantification of deceased individuals. Furthermore, the analysis of the rice's quality, including attributes such as shoulder, taste, and color, was conducted using an ANOVA test with the assistance of SPSS-25.

Organoleptic studies were conducted to evaluate the quality of rice. The experiment involved assessing the hedonic preference of cooked rice, including factors such as color, taste, and fragrance. This evaluation was conducted on rice samples that had undergone five different treatment methods. Subsequently, a sample of the rice was distributed among a cohort of 20 individuals including chefs, consumers, and nutritionists, with the objective of evaluating their respective perceptions pertaining to its visual appearance, flavor, and aroma. The evaluation of sensory attributes was conducted by the use of a numerical scale ranging from 1 to 5.

## 3. Results

### 3.1. Rice Beetle Mortality

**Figure 1:**
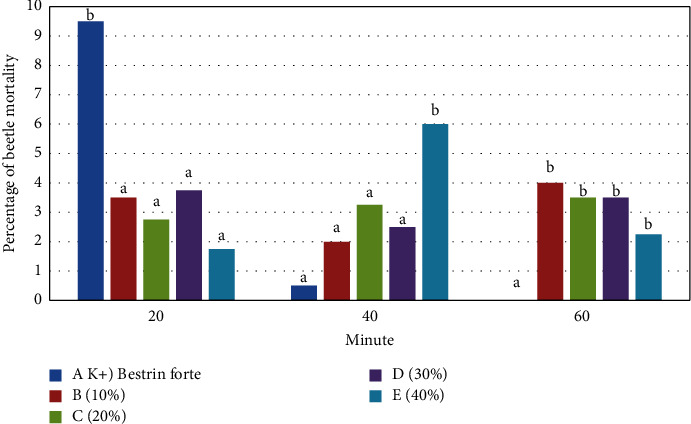
Mortality of rice beetles sprayed with *Bestrin forte* (A), and jackfruit leaf extract concentration of 10% (B), 20% concentration (C), 30% concentration (D), and 40% concentration (E) at the 20th, 40th, and 60th minutes of observation.

The mortality of rice beetles at the 20th, 40th, and 60th minutes of the results of the treatment with jackfruit leaf extract compared to the control can be seen in Figure 1.

At 20 minutes from Figure 1 shows that the mortality of rice beetles treated with *Bestrin forte* (A) as the positive control was the highest and significantly different compared to the four jackfruit leaf extract treatments, namely, 10% concentration (B), 20% concentration (C), 30% concentration (D), and 40% concentration (E), which is marked with a different letter above the bar (a, b). However, the mortality ratio of rice beetles between the four types of jackfruit leaf extract treatment was not significantly different, which is marked with the same letter (a). The mortality rate of rice beetles at 40 minutes treated with *Bestrin forte* decreased and became not significantly different from the mortality rate of jackfruit leaf extract treatment of 10% concentration, 20% concentration, and 30% concentration. Another thing happened in the treatment with the extract at a concentration of 40%, where the mortality rate was the highest and significantly different from other treatments. The 60th minute showed that the mortality of rice beetles treated with *Bestrin forte* has reached 0 (zero). This was significantly different from the four jackfruit leaf extract treatments at 10% concentration, 20% concentration, 30% concentration, and 40% concentration (Figure 1). The four jackfruit leaf extract treatments had a nonsignificantly different mortality rate for rice beetles.

**Figure 2:**
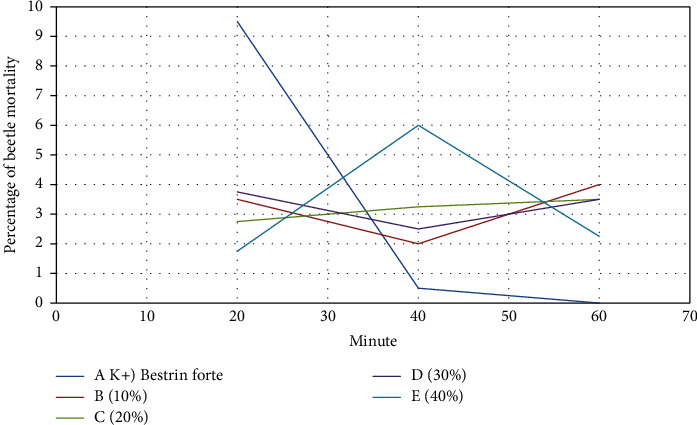
Analysis of linear regression correlation between the percentage of beetle deaths and the observation time from the 20th, 40th and 60th minutes treated with *Bestrin forte* (A), 10% concentration (B), 20% concentration (C), 30% concentration (D), and 40% concentration (E).

The mortality of rice beetles seems to be related to the duration of exposure to insecticides, being *Bestrin forte* and jackfruit leaf extract.  Figure 2 shows the relationship in each treatment *Bestrin forte* (A), 10% concentration (B), 20% concentration (C), 30% concentration (D), and 40% concentration (E). The relationship level is, respectively, 78.8%, 5.7%, 96.2%, 3.5%, and 1.1%. The relationship of these treatments describes a linear relationship with equation A is *Y* = 0.2375*x* + 12.833, B is *Y* = 0.0125*x* + 2.6667, C is *Y* = 0.0188*x* + 2.4167, D is *Y* = 0, 0063*x* + 3.5, and E is *Y* = 0.0125*x* + 2.8333.

### 3.2. Rice Quality

**Figure 3:**
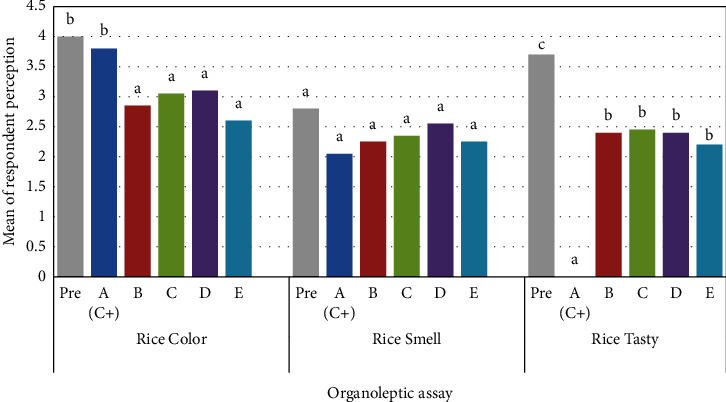
Organoleptic assessment of the color, smell, and taste of rice before being treated (pre), after being given *Bestrin forte* (A), jackfruit leaf extract at a concentration of 10% (B), a concentration of 20% (C), a concentration of 30% (D), and a concentration of 40% (E).

From testing the jackfruit leaf extract on the quality of rice regarding color, smell, and taste which have been analyzed using the application *SPSS-25* can be presented in Figure 3.

Figure 3 shows that the color of rice before being treated with *Bestrin forte* insecticide and jackfruit leaf extract was not significantly different from the color of rice treated with *Bestrin forte*. However, the color of the rice before the treatment was significantly different from the color of the rice from the extract treatment at a concentration of 10%, 20%, 30%, and 40%. The color of the rice treated with *Bestrin forte* is significantly different from the color of rice treated with jackfruit leaf extract at concentrations of 10%, 20%, 30%, and 40% concentration. The colors of the rice treated with the extract at all concentrations showed no significant differences between one another.

The smell of rice from rice before being treated and after being treated with both *Bestrin forte* and jackfruit leaf extract at the concentrations tested (10%, 20%, 30%, and 40%) showed no significant difference (Figure 3). Meanwhile, the taste of rice showed that before treatment it had the most delicious taste level and was significantly different from after being treated with *Bestrin forte* and with jackfruit leaf extract at a concentration of 10%, 20% concentration, 30% concentration, and 40% concentration. The taste of rice treated with *Bestrin forte* had the lowest taste quality compared to the taste quality of rice treated with extracts at 10%, 20%, 30%, and 40% concentrations. The quality of the taste of rice treated with jackfruit leaf extract at the four concentrations had no significant difference.

## 4. Discussion

The experimental results that have been carried out found that at the 20th minute *Bestrin Forte* (K+) had the highest mortality effect which was significantly different from the four treatments using jackfruit leaf extract at concentrations of 10%, 20%, 30%, and 40% (Figure 1). It was different in the 40th minute where a concentration of 40% had a high effect on rice beetle mortality. Meanwhile, at smaller concentrations, namely, concentrations of 10%, 20%, and 30%, they still did not have a mortality effect. *Bestrin forte* at 40 minutes had a reduced mortality effect (Figures 1 and 2). This decrease continued until it reached 0 (zero) at the 60th minute of the observation. Meanwhile, jackfruit leaf extract at a concentration of 40% still has the ability to kill beetles. These results indicate that *Bestrin forte* has a high, fast mortality rate for rice beetles, but the time is relatively short. Meanwhile, the jackfruit leaf extract has a slower, lower mortality rate for rice beetles but is quite durable. This result is in line with the study that reported that one of the weaknesses of natural pesticides is that they have a slow effect or influence on insects [18, 29, 30].

From the results of the study, it can also be stated that jackfruit leaf extract with a concentration of 40% is still not effective enough to kill rice beetles, compared to *Bestrin forte*. Therefore, it is likely to be effective if the concentration of the jackfruit leaf extract is increased. Previously, a study has reported that the concentration of lime peel extract kills rice beetles starting from a concentration of 20% to a concentration of 80% [31]. Thus, the concentration of jackfruit leaf extract is increased to 80%, and the probability of having a mortality ability against rice beetles is 3.63. The prediction is obtained from the linear regression equation which is *Y* = 0.01*X*  + 2.83 (Figure 2). The mortality rate of 3.63 or 36.3% is higher than the mortality rate achieved by *Bestrin forte* in the 20th minute which is 23%. Also, the mortality rate of 3.63 or 36.3% at 40 minutes means an increase of 21.3% from the mortality rate at 20 minutes of 15%.

The mortality effectiveness of jackfruit leaf extract at a concentration of 40% occurred in the 40th minute. This means that the content of bioactive compounds such as flavonoids from jackfruit leaf extract functions optimally in that minute. The results of the study show that flavonoids function to interfere with the nervous system of insects [32–34]. This opinion is reinforced by observations showing that dead rice beetles have signs of staggering, trembling legs, and sleepy eyes (unpublished data). These characteristics agree with a study reporting that flavonoids work as inhibitors in the respiratory system [35]. Flavonoids attack several nervous organs in several vital organs of insects, causing a weakening of the nervous system and respiratory system and resulting in death. Apart from flavonoids, other compounds contained in jackfruit leaf extract which are thought to have an effect on the mortality of rice beetles are saponins and tannins. This conjecture is based on the opinion that tannin compounds can cause digestive enzyme activity and decrease absorption and interfere with the body's metabolic processes [36]. In addition, tannins can reduce the activity of digestive enzymes (protease and amylase) and interfere with intestinal protein activity, so that it will experience nutritional disorders [37]. The workings of saponins are as stomach poisons and inhibit the action of enzymes [38].

The mortality of rice beetles is related to the duration of exposure by jackfruit leaf extract. From the data in Figure 2, it shows that the *Bestrin Forte* (A) treatment has an effect value of 78.94% on rice beetle mortality. Furthermore, treatment with a concentration of 10% (B) was 5.77% on the mortality of rice beetles. Then, with the treatment concentration of 20% (C) with a correlation value of 0.981, the results that have been obtained are included in the very strong category. The effect value is 96.2%, which means that jackfruit leaf extract affects 96.2% of rice beetle mortality. Treatment with a concentration of 30% (D) with a correlation value of 0.188, the results obtained were included in the very low category and an effect value of 3.5%, which means that jackfruit leaf extract affects 3.5% of rice beetle mortality. Finally, with a treatment concentration of 40% (E) with a correlation value of 0.107, the results obtained are included in the very low category and the effect value is 1.1%.

From the description mentioned above, there is an interesting fact from the study that extracts with high concentrations (40%) have faster mortality compared to extracts with lower concentrations. These results indicate that jackfruit leaf extract has high levels of bioactive; flavonoids, saponins, and tannins. This is a line white a study revealed that the increase in concentration is directly proportional to the increase in the toxic substance so that the killing power is higher [39]. In addition, it is suspected that high concentrations have lower moisture compared to extracts from low concentrations. Air humidity comes from the evaporation of water from the extract. This is based on an opinion expressing that lower humidity can cause high mortality [40].

The organoleptic analysis carried out in the study aims to determine the level of consumer preference for color, smell, and taste in rice that is given jackfruit leaf extract. The rice color data obtained that before the treatment was not significantly different from the *Bestrin Forte* treatment. This means that *Bestrin Forte* did not change the color of the rice. However, prior to treatment it was significantly different from extract treatments (10%, 20%, 30%, and 40% concentration). These results illustrate that jackfruit leaf extract concentrations resulted in a significant change in the color of the rice from the color of the rice before being treated. The color of rice treated with *Bestrin forte* was the same as the color of normal rice being bone white, while the color of rice treated with 10% jackfruit leaf extract was light green, then the color of rice treated with 20% jackfruit leaf extract was greenish white. The color of rice treated with jackfruit leaf extract 30% is pale white, and finally the color of rice treated with 40% jackfruit leaf extract is yellowish white. The change in the color of the rice that had been treated, and it indicated that the jackfruit leaf extract affected the color of the rice. The change depends on the different concentrations. The results mean that the compounds contained in the jackfruit leaf extract are of different levels, and then interact with rice so that the color of the rice becomes different. The compounds that change the color of the rice are thought to be pigments from jackfruit leaf extract. This is based on a study reveal that changes in rice color are caused by the blending of the color pigments from the leaves with the rice [41]. Changes in the color of rice between rice from before and after treatment resulted in a decrease in the quality of the color of rice. This research is in line with a previous research conducted which stated that lime leaf extract reduced the color quality of rice [41].

Odor is one of the parameters in testing sensory properties (organoleptic) using the sense of smell. On rice before and after extract treatment showed that the quality of the smell of rice after being given treatment did not decrease. There was no decrease in the quality of the smell of the rice because when the rice was put into the plastic sample was cold so there was no air deposition in the plastic sample given to the respondent. As revealed by a study that reported that the cause of the decrease in the quality of the smell of rice is caused by rice that is still hot which is put directly into the container [41].

Taste is one of the factors that can determine whether a product can be accepted or not by consumers. The rice taste data obtained was a significant difference between before and after treatment. However, the taste of rice treated with extract has almost the same taste. The taste of rice treated with jackfruit leaf extract 10%, 20%, 30%, and 40% had an unpleasant taste (Figure 3). The scoring value of the rice before and after treatment indicated that the taste quality of the rice after being given treatment decreased from quite tasty to less tasty. The decrease in the taste of rice after treatment is thought to be due to the presence of compounds in jackfruit leaves which can cause a bitter taste, causing an unpleasant taste.

## 5. Conclusion

The use of *Artocarpus heterophyllus* leaf extract has shown a notable impact on the death rate of rice beetles. Furthermore, this extract demonstrated the ability to preserve the olfactory characteristics of rice, hence maintaining its overall quality. Nevertheless, it is unable to uphold the standard of the rice in relation to its visual appearance and flavor. The concentration of 40% was shown to be the most efficacious in terms of rice beetle eradication while utilizing jackfruit leaf extract.

## Data Availability

The data used to support the study are available from the corresponding author upon request. https://docs.google.com/spreadsheets/d/1W44ZKbUL9H1i0iu4KXU0fINNUxUDG7DZ/edit#gid=1404597595.
